# Induction of Immune Responses and Phosphatidylserine Exposure by TLR9 Activation Results in a Cooperative Antitumor Effect with a Phosphatidylserine-targeting Prodrug

**DOI:** 10.7150/ijbs.81683

**Published:** 2023-05-11

**Authors:** Jen-Chih Tseng, Jing-Xing Yang, Chia-Yin Lee, Chen-Fu Lo, Yi-Ling Liu, Mingzi M. Zhang, Li-Rung Huang, Ko-Jiunn Liu, Chien-Chia Wang, Chi-Ying F. Huang, Yi-Ren Hong, Lun K. Tsou, Tsung-Hsien Chuang

**Affiliations:** 1 Immunology Research Center, National Health Research Institutes, Zhunan, Miaoli 35053, Taiwan.; 2 Institute of Biotechnology and Pharmaceutical Research, National Health Research Institutes, Zhunan, Miaoli, 35053, Taiwan.; 3 Institute of Molecular and Genomic Medicine, National Health Research Institutes, Zhunan, Miaoli, 35053, Taiwan.; 4 National Institute of Cancer Research, National Health Research Institutes, Zhunan, Miaoli 35053, Taiwan.; 5 Department of Life Sciences, National Central University, Zhongli District, Taoyuan City 32001, Taiwan.; 6 Institute of Biopharmaceutical Sciences, College of Pharmaceutical Sciences, National Yang Ming Chiao Tung University, Taipei 11221, Taiwan.; 7 Graduate Institute of Medicine, College of Medicine, Kaohsiung Medical University, Kaohsiung 80708, Taiwan.

**Keywords:** Cancer immunotherapy, CpG-oligodeoxynucleotide, Immunogenic cell death, Phosphatidylserine-targeting prodrug, Toll-like receptor, Topoisomerase I inhibitor

## Abstract

Head and neck cancer is a major cancer type, with high motility rates that reduce the quality of life of patients. Herein, we investigated the effectiveness and mechanism of a combination therapy involving TLR9 activator (CpG-2722) and phosphatidylserine (PS)-targeting prodrug of SN38 (BPRDP056) in a syngeneic orthotopic head and neck cancer animal model. The results showed a cooperative antitumor effect of CpG-2722 and BPRDP056 owing to their distinct and complementary antitumor functions. CpG-2722 induced antitumor immune responses, including dendritic cell maturation, cytokine production, and immune cell accumulation in tumors, whereas BPRDP056 directly exerted cytotoxicity toward cancer cells. We also discovered a novel function and mechanism of TLR9 activation, which increased PS exposure on cancer cells, thereby attracting more BPRDP056 to the tumor site for cancer cell killing. Killed cells expose more PS in tumor for BPRDP056 targeting. Tumor antigens released from the dead cells were taken up by antigen-presenting cells, which enhanced the CpG-272-promoted T cell-mediated tumor-killing effect. These form a positive feed-forward antitumor effect between the actions of CpG-2722 and BPRDP056. Thus, the study findings suggest a novel strategy of utilizing the PS-inducing function of TLR9 agonists to develop combinational cancer treatments using PS-targeting drugs.

## Introduction

Head and neck cancers are among the most common cancer types with high mortality rates. Head and neck squamous cell carcinoma (HNSCC) account for >90% head and neck cancer cases, often characterized by an immunosuppressive microenvironment, consistent with the fact that only <20% patients respond to therapy with immune checkpoint inhibitors. Currently, standard treatments for patients with HNSCC include radiation, chemotherapy, and cetuximab. However, patients with cancer suffer from poor quality of life due to the disease and treatment; therefore, highly effective and satisfactory therapeutic strategies are desired 1-3.

Toll-like receptors (TLRs) are a family of pattern recognition receptors that help detect microbial pathogens to initiate host responses to infections. Of the 13 TLRs (TLR1-13) identified in mammals, 10 (TLR1-10) are expressed in humans. TLR9 belongs to a subfamily comprising TLR3, TLR7, TLR8, and TLR9. Distinct from other human TLRs expressed on cell surfaces, these four TLRs are located in intracellular vesicles, primarily in endolysosomes 4, 5. The natural TLR9 ligand is microbial unmethylated CpG-DNA. The ligand ligation of TLR9 activates nuclear factor (NF)-κB and interferon regulatory factors (IRFs), thereby producing inflammatory cytokines and type I interferons (IFNs), respectively 6, 7. The proximal signal molecule for TLR9 signal transduction is the myeloid differentiation primary response 88 (MyD88). On activation, MyD88 is recruited to TLR9, forming a complex with interleukin (IL)-1 receptor-associated kinase-1 (IRAK-1), IRAK-4, and tumor necrosis factor (TNF)-associated factor 6 (TRAF6) and transforming growth factor-β-associated kinase 1 (TAK1) activation. This cascade activates NF-κB, responsible for the transcription of genes for proinflammatory cytokines, including TNF-α, IL-1, IL-6, and IL-12. The dendritic cell (DC) is a major cell type for type I IFN production in response to TLR9 activation. In these cells, the MyD88, IRAK1, IRAK4, and TRAF6 complex activates IRF-7 phosphorylation and translocation into the nucleus for type I IFNs transcription 8-11.

Synthetic CpG-oligodeoxynucleotides (CpG-ODNs) mimic CpG-DNA's function in TLR9 activation. Distinct from natural CpG-DNAs containing a phosphodiester backbone, CpG-ODNs include a phosphorothioate backbone that increases resistance to nucleases 12, 13. TLR9 activation via CpG-ODNs administration induces an early innate immune response for priming the subsequent adaptive immune responses. Innate immune and B cells are activated during the early phase for antigen presentation and cytokine production. The produced cytokines promote Th1 CD4+ T-cell polarization, thereby expanding antigen-specific CD8+ T cells 14-17. As these immune responses play a crucial role in cancer cell eradication, the antitumor effect of CpG-ODNs has been investigated as both monotherapies and combinational therapies 18, 19.

Phosphatidylserine (PS) is an essential bilayer cell membrane component. It normally exists in the inner leaflet of the cell; however, it can be externalized to the external leaflet in response to cell injury or apoptosis. The PS exposed on apoptotic cells act as an engulfment signal for phagocytosis by phagocytic cells. Recently, PS exposure has also been reported in nonapoptotic forms 20,21. In tumors, PS exposure is significantly increased on tumor cell surface, serving as a targeting marker for diagnosis and therapy. Consequently, PS-targeting agents have been developed, including natural products, monoclonal antibodies, antibody-drug conjugations, and liposomal carriers. Several of these are under investigation in clinical trials as potential monotherapies or combination drugs for various cancers 22, 23. Zinc (II)-dipicolylamine (Zn-DPA) reportedly binds specifically to PS 24, 25.

SN38 (7-ethyl-10-hydroxycamptothecin) is a topoisomerase I inhibitor with cytotoxicity toward cancer cells. This agent cannot be directly administered to patients owing to its toxicity and aqueous solubility features; therefore, chemical modification is required to deliver it more explicitly to tumors 26, 27. BPRDP056 is a novel PS-targeting antitumor drug candidate generated by linking Zn-DPA with SN38. It reportedly suppresses the growth of several different types of tumors in cancer animal models 28. Nevertheless, to the best of our knowledge, its suppressing effect on head and neck cancers has not yet been investigated. CpG-2722 is a CpG-ODN exhibiting TLR9-mediated immune-stimulatory activities for multiple species 29, 30. Herein, we utilized an orthotopic syngeneic mouse model of HNSCC to investigate the mechanism and function of combined CpG-2722 and BPRDP056 therapy to treat head and neck cancers. The results revealed that BPRDP056 displayed cytotoxicity in cancer cells. TLR9 activation by CpG-2722 activated immune responses and PS exposure in tumors. CpG-2722 and BPRDP056 administration alone inhibited tumor growth. The combination treatment with both agents further produced a cooperative effect on tumor growth suppression.

## Materials and methods

### Animal care

All animal experiments were approved by the Institutional Animal Care and Use Committee of the National Health Research Institute, Taiwan. Both male and female mice were used after randomization to the different experimental groups. These mice were maintained and handled following the stated guidelines.

### Chemicals, reagents, and antibodies

CpG-2722 was purchased from Integrated DNA Technologies, Inc. Small molecule compound, BPRDP056, BPRDP060, and SN38 were synthesized and dissolved for treatments as reported previously 28. Rat antimouse CD8 antibody (clone: 4SM15, Cat. No. 14-0808-82) was purchased from Invitrogen. Rabbit anti-PS antibody (Cat. No. PAB881Ge01) was purchased from Cloud-Clone Corp. Rat anti-mouse IFN-γ antibody (MAB485) for neutralizing was purchased from R&D. Rat anti-mouse TNF-related apoptosis-inducing ligand (Trail) neutralizing antibody (Cat. No. 109315) and hamster anti-mouse TNF-α neutralizing antibody (Cat. No. 506110) were purchased from BioLegend. Mouse IFN-α, and the Trail enzyme-linked immunosorbent assay (ELISA) kit were purchased from R&D. Rabbit anti-TrailR2 antibody (Cat. No. ab8416) was purchased from Abcam. Trizol reagent and the SuperScriptTM IV kit were purchased from Invitrogen. The SYBR® Green PCR kit was purchased from Qiagen.

### Cell culture

SAS is a human tongue squamous cell carcinoma (SCC) cell line 31. OECM1 is a human gingival squamous carcinoma cell line harboring a p53 missense mutation 32. M11-1-2 and NHRI-HN1 are mouse tongue SCC cell lines established previously 33. SAS, M11-1-2, and NHRI-HN1 were maintained in Dulbecco's modified Eagle medium (complete medium) containing 10% fetal bovine serum (FBS) (Hyclone), 2 mM L-glutamine, 1% antibiotic-antimycotic, and 10 mM HEPES (4-(2-hydroxyethyl)-1-piperazineethanesulfonic acid). OECM1 cancer cells were maintained in RPMI complete medium containing 10% FBS, 1% antibiotic-antimycotic, and 10 mM HEPES and cultured at 37°C in a 5% CO_2_ incubator.

### Mouse splenocytes preparation

Mouse splenocytes were isolated from 6- to 8-week-old C57BL/6J mice (National Laboratory Animal Center, Taiwan). Mouse spleen fragments were pounded using a syringe plunger; single cells were collected after passing them through a 40-μm nylon cell strainer (BD FalconTM) and centrifuged at 1500 rpm for 5 min. Red blood cells (RBCs) were removed by resuspending the cell pellet in RBC lysis buffer at room temperature for 2 min, and the lysis reaction was terminated by adding 30 ml phosphate-buffered saline (PBS). The isolated splenocytes were cultured in RPMI 1640 complete medium at 37°C in a 5% CO_2_ incubator.

### Murine bone marrow-derived dendritic cells preparation

Mouse bone marrow-derived dendritic cells (BMDCs) were prepared from C57BL/6J mice. Bone marrow cells were flushed from the femur and tibia with PBS and resuspended in RPMI-1640 complete medium containing 10% FBS, 2 mM L-glutamine, 1% antibiotic-antimycotic, 1% MEM nonessential amino acid solution, 1 mM sodium pyruvate, 10 mM HEPES supplemented with 100 ng/mL FMS-like tyrosine kinase 3 ligand (Flt3-L) (PeproTech) and 5 ng/mL granulocyte-macrophage colony-stimulating factor (GM-CSF) (PeproTech). On days three and six, a complete medium supplemented with 100 ng/mL Flt3-L and 5 ng/mL GM-CSF was added for further culture. On day nine, immature DCs were seeded at 2 × 10^6^ cells/well of a noncoated six-well plate and stimulated with CpG-2722 at 5 μg/ml for 24 h.

### Flow cytometry

BMDCs maturation status was assessed by fluorescence-activated cell sorting (FACS). Briefly, BMDCs were harvested and rinsed twice with ice-cold PBS containing 2% FBS and spun at 1500 rpm for 5 min at 4°C. The cells were surface-antibody stained for CD11c (Cat.No. 117308, BioLegend), CD40 (Cat. No. 12-0401-82, Invitrogen), CD80 (Cat. No. 17-0801-82, Invitrogen), and CD86 (Cat. No. 25-0862-82, Invitrogen) in ice-cold PBS containing 2% FBS for 30 min. After washing twice with PBS containing 2% FBS, these surface molecules expression levels on BMDCs were acquired on a FACS Canto II and analyzed using FlowJo software. To analyze immune cells in tumors, tumor samples were incubated in gentle collagenase/hyaluronidase in DMEM for enzymatic dissociation. After overnight incubation, cells were collected after passing them through a 70-μm nylon cell strainer and centrifuged at 1500 rpm for 5 min. The cells were surface-antibody stained as indicated in ice-cold PBS containing 2% FBS for 30 min. After washing twice with PBS containing 2% FBS, the immune cells composition in tumors were acquired on a FACS Canto II and analyzed using FlowJo software.

### Cell cytotoxicity assay

Mouse splenocytes and cancer cells were seeded onto 96 well plates at 10^6^ and 3 × 10^3^ in 100 μl/well, respectively. The next day, splenocytes and cancer cells were treated with the CpG-2722, BPRDP056, BPRDP060, and SN38 as indicated. Cell cytotoxicity was determined using a CellTiter 96 Aqueous One Solution Cell Proliferation Assay kit (Promega) following the manufacturer's protocol.

### RNA isolation and reverse transcription-quantitative PCR (RT-qPCR) analyses

Total RNA from cells were isolated using the illustra^TM^ RNAspin Mini kit (GE Healthcare). Total RNA from tumors was isolated using Trizol reagent following the manufacturer's protocol. RNA sample's reverse transcription was performed using the SuperScript IV First-Strand Synthesis System (Invitrogen). Gene expression levels induced by CpG-2722 or BPRDP056 were analyzed using the QuantiNovaTM SYBR Green PCR kit (Qiagen) for qPCR analyses and gene specific primers (Table S1). β-actin expression level served as the loading control.

### Syngeneic orthotopic animal model of head and neck cancer

Two million NHRI-HN1 cells were mixed with Matrigel (BD Biosciences) at a 1:1 ratio to a total volume of 100 μl per mouse. The NHRI-HN1 cells were intramucosally injected into the 6- to 8-week-old C57BL/6J mice through a side of the buccal region to grow the tumor. When the tumor size was approximately 100 mm^3^, the mice were intratumorally injected with the indicated amount of CpG-2722, BPRDP056, and their combination every 4 days. All groups contained five mice and five tumors. The tumor volume was measured using the formula: Volume = length × (width)^ 2^ × 0.5.

### ELISA

Mouse splenocytes or tumor tissue lysates culture medium was collected. TNF-α, IFN-α, IFN-γ, and Trail production levels were determined using ELISA kits following the manufacturer's protocol.

### Immunohistochemistry

Paraffin-embedded tumors were sectioned into 5-μm tissue slides. These tissue slides were rehydrated with graded ethanol to PBS concentrations, and endogenous peroxidase was blocked with 3% hydrogen peroxide for 5 min. For PS, Trail, and CD8 staining, the primary antibody against PS or CD8 was used at a 1:50 dilution, and the Trail antibody was used at a 1:25 dilution. The tissue slides were incubated with specific antibodies at room temperature for 1 h. Tissue sections were then incubated with horseradish peroxidase-conjugated secondary antibody at room temperature for 30 min after washing with PBS solution. Detection was processed using the Discovery XT automated IHC/ISH slide staining system (Ventana Medical System, Inc., Tucson) using an ultraView Universal DAB Detection Kit (Ventana Medical System, Inc., Tucson), according to the manufacturer's instructions. PS-positive areas percentage and the number of CD8-positive cells were calculated using ImageJ (RRID:SCR-003070).

### Phosphatidylserine exposure analysis

The condition medium collection following mouse splenonocytes were stimulated with or without 5 μg/ml of CpG-2722 for 48 h. NHRI-HN1 cells were treatment with DMEM containing 25% condition medium for 8 h. The PS exposure were stained by annexin V conjugated APC antibody (Biolegend) at room temperature for 15 min, and the NHRI-HN1 PS exposure level were acquired on a FACS Canto II and analyzed using FlowJo software.

### Statistical analysis

Data are expressed as mean ± SEM. All groups were from three or more independent experiments as indicated. Statistical analyses were performed using Student's t-test. Differences were considered significant when the *p* value was less than 0.05. Statistically significant *p* values are abbreviated as follows: *, *p* < 0.05; **, *p* < 0.01; ***, *p* < 0.001.

## Results

### Cytotoxic effect of BPRDP056 and CpG-2722

**Figure 1:**
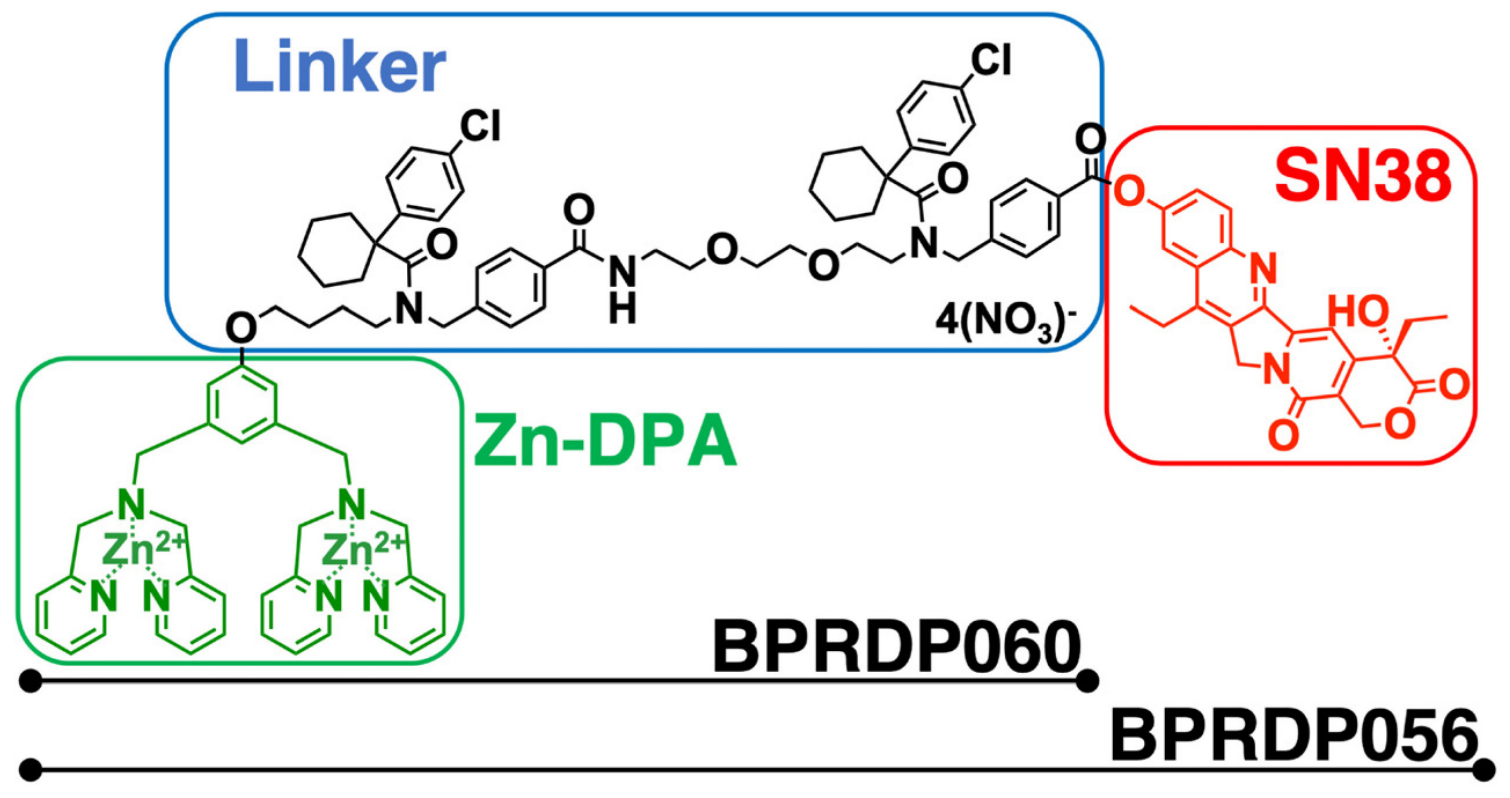
** Structural features of BPRDP056. BPRDP056 is a phosphatidylserine (PS)-targeting prodrug of SN38.** BPRDP056 contains three moieties: SN38 for tumor-cell killing; Zn-DAP for PS targeting; and a linker between SN38 and Zn-DAP. BPRDP060 contains the linker and Zn-DAP regions of BPRDP056. These compounds were prepared according to the previous report 28. Briefly, by coupling the pegylated linker that contains the cyclohexyl-para-chlorophenyl functional groups to the Zn-DPA, the precursor of BPRDP060 was first prepared. Condensation between SN38 and the precursor of BPRDP060 allowed the synthesis of BPRDP056 precursor. Incubation of each precursor with two equivalents of zinc nitrate resulted in the formation of Zn-DPA conjugates BPRDP060 and BPRDP056.

**Figure 2:**
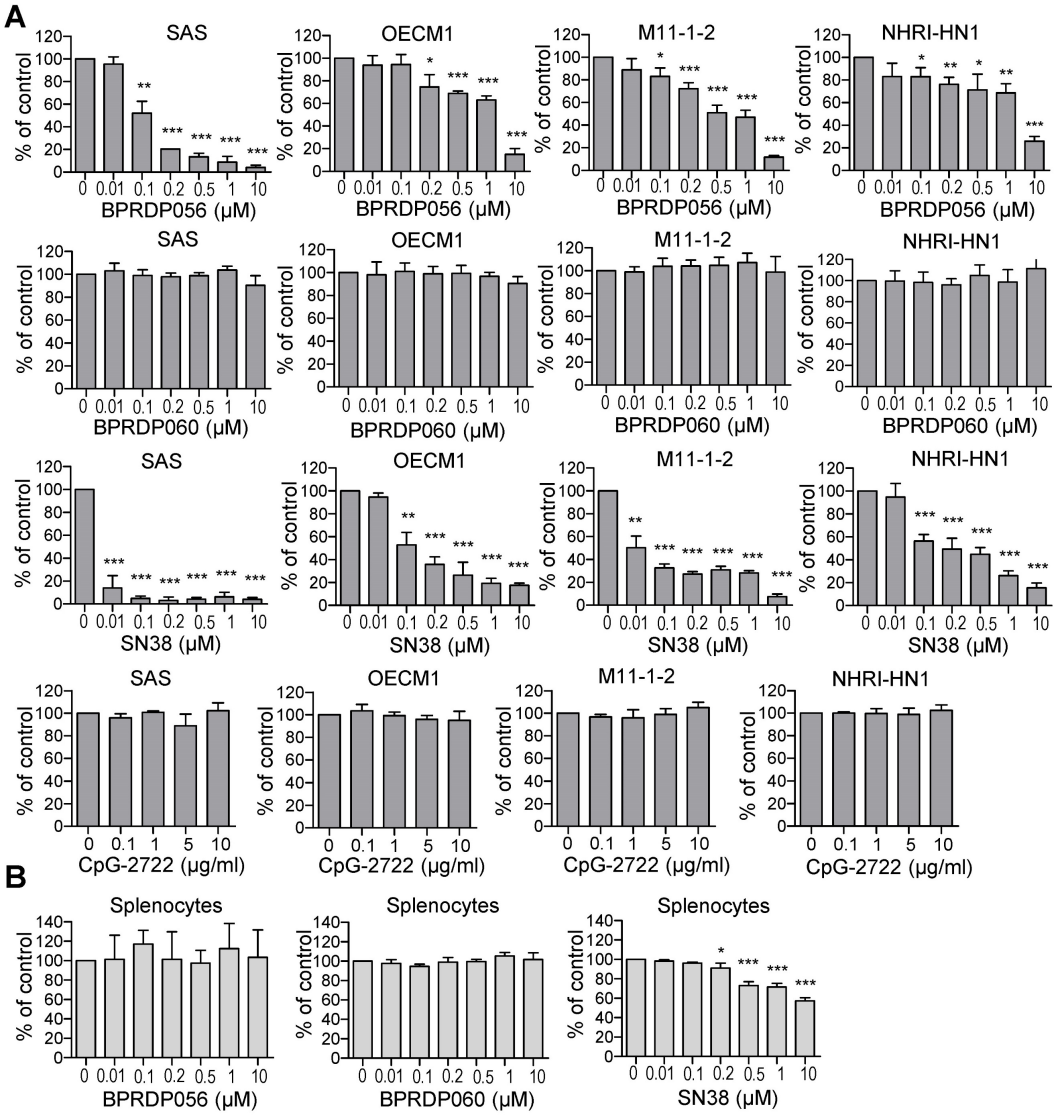
** Cytotoxic effects of BPRDP056 and CpG-2722. (A)** Human tongue squamous cell carcinoma SAS cells, human gingival squamous carcinoma OECM1cells, and mouse tongue squamous cell carcinoma M11-1-2 and NHRI-HN1 cells, and **(B)** mouse splenocytes were treated with different concentrations of BPRDP056, BPRDP060, SN38, or CpG-2722 for 72 h, as indicated. Cell cytotoxicity was then determined using the CellTiter 96 aqueous assay. Data represent the mean ± SEM (n = 3 independent experiments). *, **, and *** represent statistically significant differences; *p* < 0.05, *p* < 0.01, and *p* < 0.001, respectively, compared with the control vehicle.

BPRDP056 is a prodrug generated by conjugating BPRDP060 to SN38. The BPRDP060 comprises Zn-DPA for PS targeting and a linker region to bridge SN38 and Zn-DPA (Figure 1) 28. To investigate the feasibility of using BPRDP056 alone and in combination with CpG-2722 on head and neck cancers, we first examined the cytotoxicity of BPRDP056 toward several different lines of head and neck cancer cells, including the human SAS and OECM1 lines as well as the mouse M11-1-2 and NHRI-HN1 lines. BPRDP056 exerted different cytotoxicity toward these cells. Human SAS was more sensitive than OECM1, and mouse M11-1-2 was more sensitive than NHRI-HN1 to BPRDP056 cytotoxicity. The cytotoxicity of the different parts of BPRDP056 and CpG-2722 was also investigated. When compared with BPRDP056, SN38 exhibited more powerful cytotoxicity. However, the degree of cytotoxicity of both BPRDP056 and SN38 was similar across the four analyzed cell lines. Conversely, the cytotoxicity of BPRDP060 and CpG-2722 were not observed under the experimental conditions (Figure 2A). These findings suggest that SN38 is the moiety responsible for the cytotoxicity of BPRDP056 on these head and neck cancer cells. The cytotoxicities of BPRDP056, BPRDP060, and SN38 were further assessed on mouse splenocytes. Some cytotoxicity of SN38 was observed when its concentration reached 0.2 μM. Nevertheless, the cytotoxicity of BPRDP056 on these cells was not observed even at 10 μM (Figure 2B), suggesting that compared with the head and neck cancer cells, splenocytes were less sensitive to BPRDP056.

### Immunostimulatory effect of CpG-2722 and BPRDP056

**Figure 3:**
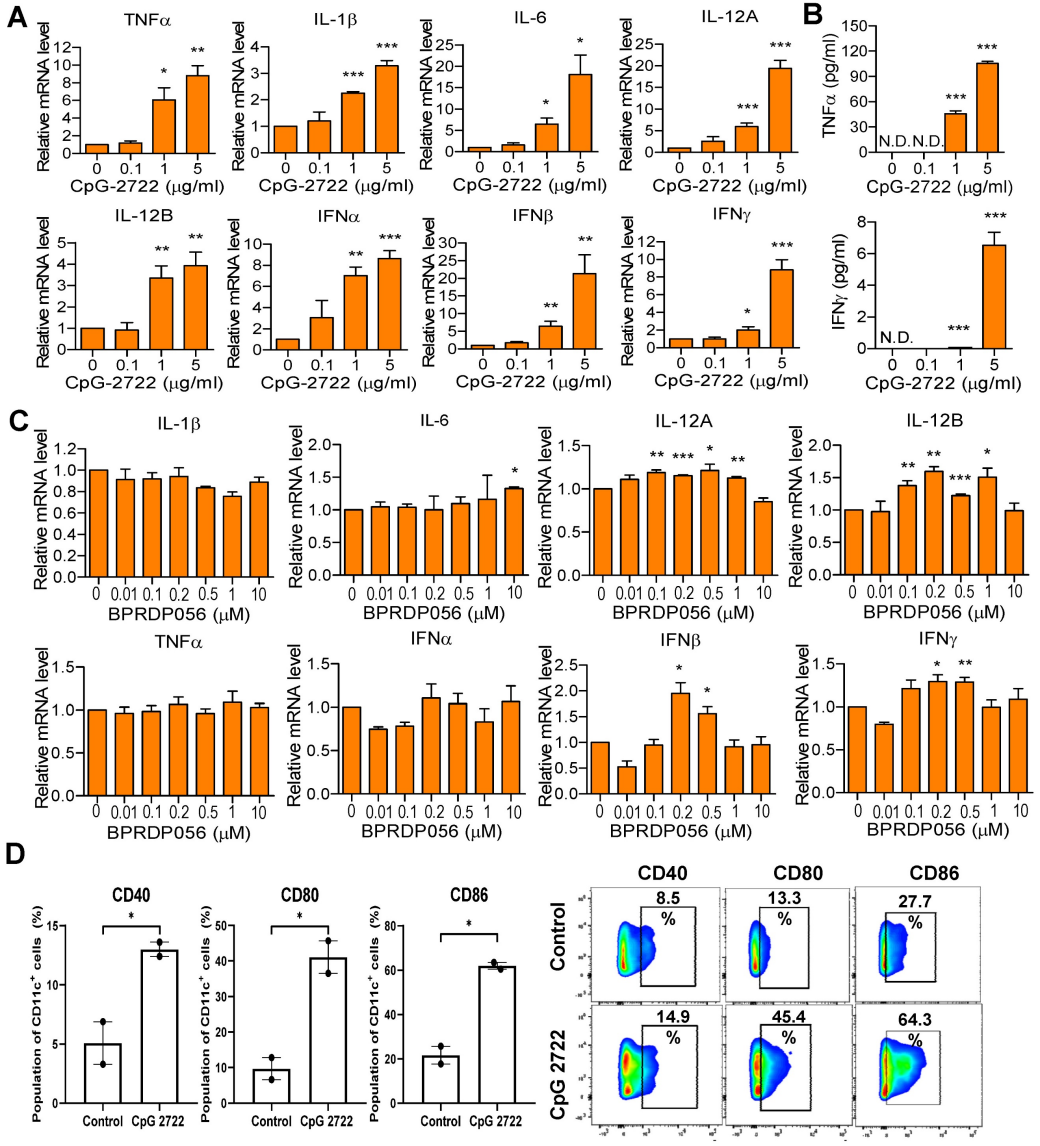
** Immune-stimulatory activities of CpG-2722 and BPRDP056.** Mouse splenocytes were treated with different concentrations of CpG-2722 as indicated. **(A)** After 4 h, expressions of cytokines were analyzed by RT-qPCR. The expression level of actin was used as a loading control. **(B)** After 24 h, cytokines secreted into the cell culture medium were measured by ELISA, as indicated. **(C)** Mouse splenocytes were treated with different concentrations of BPRDP056 as indicated. After 4 h, expressions of cytokines were analyzed by RT-qPCR. **(D)** Bone marrow-derived dendritic cells (BMDCs) were treated with 5 μg/ml of CpG-2722 for 24 h. Left panels: expressions of cluster of differentiation (CD)40, CD80, and CD86 were analyzed by flow cytometry. Right panels: a set of representative histograms for flow cytometric analyses. Data represent the mean ± SEM (n = 3; independent experiments). *, **, and *** represent statistically significant differences; *p* < 0.05, *p* < 0.01, and *p* < 0.001, respectively, compared with the control.

The immunostimulatory activities of CpG-2722 and BPRPD056 were compared. Mouse splenocytes were treated with CpG-2722, and the expression of cytokines was analyzed via RT-qPCR. CpG-2722 significantly induced various cytokines expression, including that of TNF-α, IL-1β, IL-6, IL-12A, IL-12B, IFN-α, IFN-β, and IFN-γ (Figure 3A). Among these, TNF-α and IFN-γ are signature Th1 immune response cytokines. Additionally, TNF-α displays cytostatic and cytotoxic effects against cancer cells 34, 35. Therefore, the production of these two cytokines was verified via enzyme-linked immunosorbent assay (ELISA). Consistent with their increased gene expression profiles, these two cytokine productions increased in parallel with CpG-2722 concentrations (Figure 3B). Next, BPRDP056 immunostimulatory activities were investigated. Compared with CpG-2722, BPRDP056 elicited a milder immune response. Weak cytokine induction, including that of IL-6, IL-12A, IL-12B, IFN-β, and IFN-γ, was observed when the BPRDP056 concentration increased to certain levels. The immunostimulatory activity disappeared on further increase in concentration (Figure 3C). DCs comprise major antigen-presenting cells 36, 37; thus, CpG-2722 activity on DC was investigated. CpG-2722 promoted the expression of CD40, CD80, and CD86, markers for DC maturation and costimulatory molecules of DCs for T-cell activation (Figure 3D) 36, 37. These results indicate CpG-2722 immune-stimulatory activities and suggested distinct functional profiles for the antitumor effect of CpG-2722 and BPRDP056.

### Tumor suppressing effect of BPRDP056 on HNSCC

**Figure 4:**
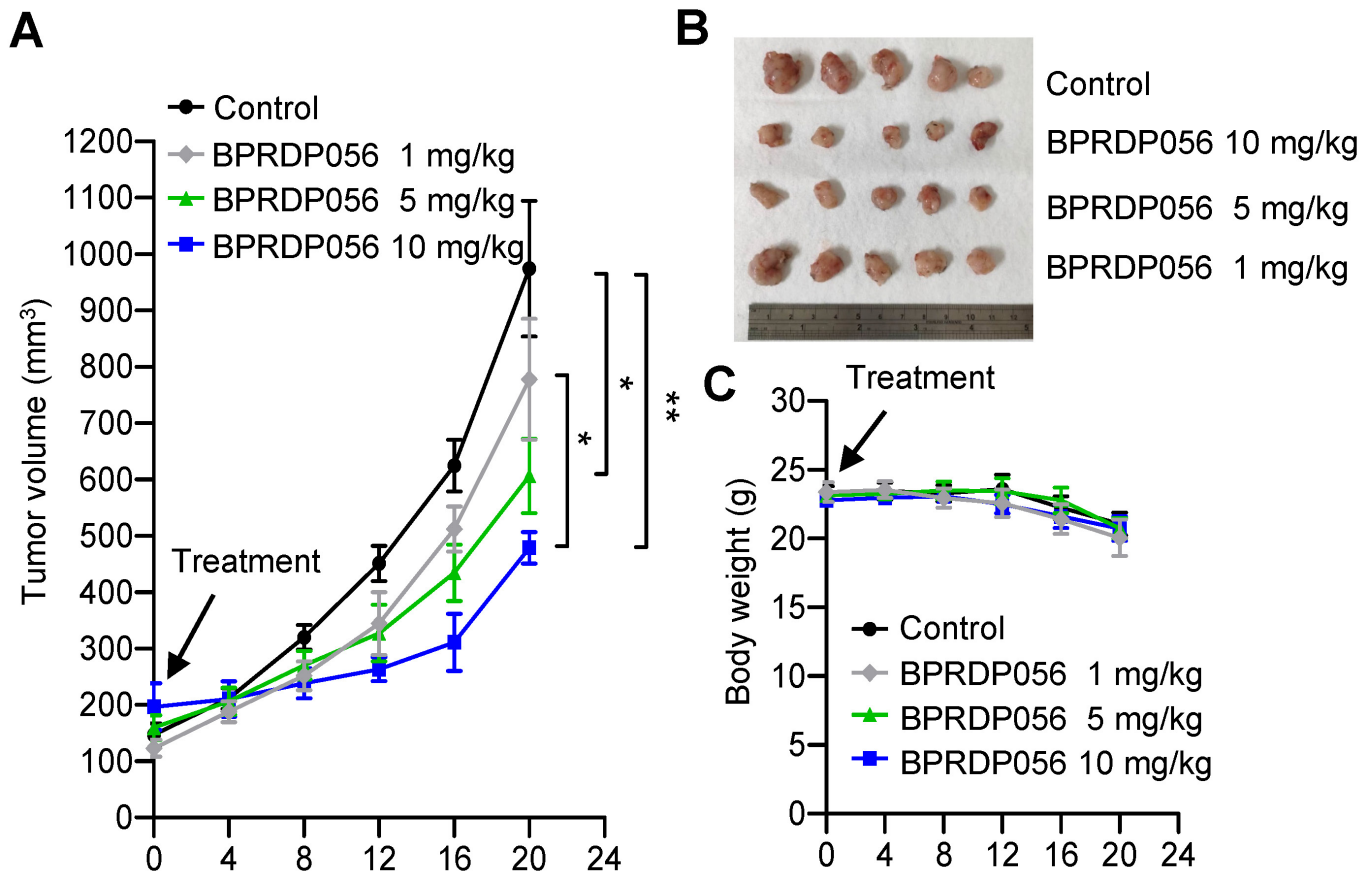
** Inhibitory effects of BPRDP056 on head and neck squamous cell carcinoma (HNSCC).** C57BL/6J mice were orthotopically injected with 2 × 10^6^ NHRI-HNC1 cells to establish HNSCC. When the tumors reached approximately 100 mm^3^, the mice were intratumorally injected with a control vehicle and different doses of BPRDP056 every 4 days, as indicated. **(A)** The tumor size was measured every 4 days (each group contained five mice and five tumors). **(B)** The endpoint of tumor growth in every group. **(C)** The body weight of each mouse was measured every 4 days (n = 5). Data represent the mean ± SEM. *, **, and *** represent statistically significant differences; *p* < 0.05, *p* < 0.01, and *p* < 0.001, respectively, between different groups, as indicated.

The head and neck cancer cell line, NHRI-HN1, was established from C56BL/6J-derived oral squamous cell carcinoma cells to develop an orthotopic syngeneic cancer animal model to study the cancer immunobiology of head and neck cancers 33. We investigated the antitumor activity of the BPRDP056 with this HNSCC animal model. The NHRI-HN1 cells (2 × 10^6^ cell/mouse) were injected into the mice through the side of the buccal region to develop orthotopic tumors. When the size of the tumors reached ~100 mm^3^, the mice were intratumorally injected with 1, 5, or 10 mg/kg/mouse BPRDP056 every 4 days. A dose-dependent effect of the BPRDP056 on HNSCC growth suppression was observed (Figure 4). The dose of 10 mg/kg BPRDP056/mouse exhibited a higher antitumor effect than the other two doses, although the tumors continued to grow. Therefore, this BPRDP056 dose was used in the subsequent studies to investigate its combinational effect with CpG-2722.

### Cooperative effect of CpG-2722 and BPRDP056 on HNSCC suppression

**Figure 5:**
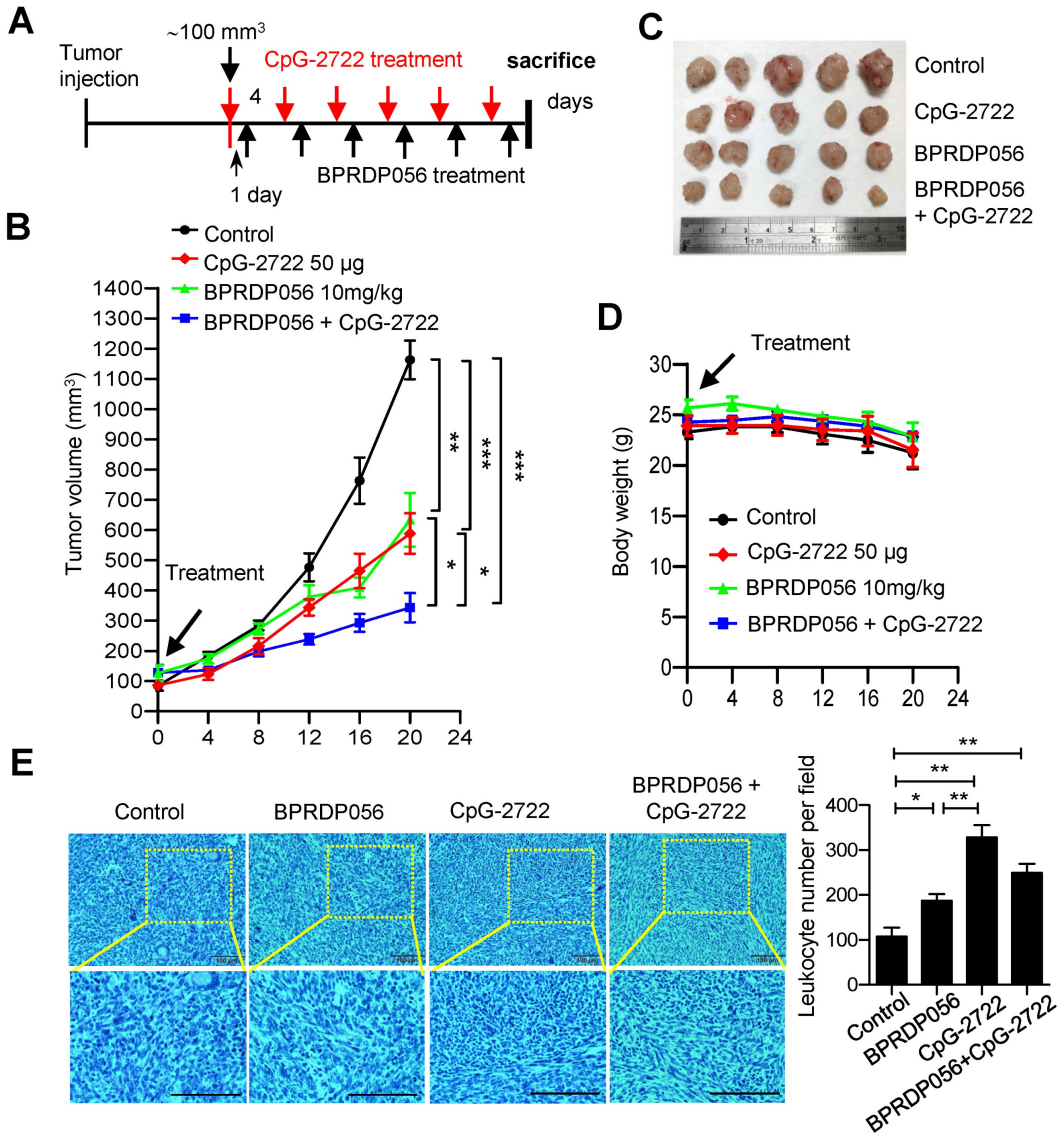
** Cooperative effect of BPRDP056 and CpG-2722 on suppressing the growth of head and neck squamous cell carcinomas (HNSCC). (A)** C57BL/6J mice were orthotopically injected with 2 × 10^6^ NHRI-HNC1 cells to grow HNSCC. When the tumors reached approximately 100 mm^3^, the mice were intratumorally injected with a control vehicle, 10 mg/kg BPRDP056 or 50 μg CpG-2722 alone and in combination every 4 days with the injection of CpG-2722 one day ahead of the BPRDP056 injection, as illustrated. **(B)** The growth of tumors was measured (n = 5). **(C)** The endpoint of tumor growth in every group. **(D)** The body weight of each mouse was measured (n = 5). **(E)** Tumor samples were visualized by H&E staining for leukocyte infiltrations. Scale bar represents 100 μm. Leukocyte infiltrations were counted using ImageJ. Data represent the mean ± SEM. *, **, and *** represent statistically significant differences; *p* < 0.05, *p* < 0.01, and *p* < 0.001, respectively, between different groups, as indicated.

To investigate the suppression of HNSCC via combinational therapy involving BPRPD056 and CpG‑2722, the mice were continuously intratumorally injected with CpG-2722 and BPRPD056 alone or in combination every 4 days following the schedule shown in Figure 5A when the tumors reached ~100 mm^3^. These mice were monitored for tumor growths (Figure 5B), euthanized at the end of the CpG-2722 and BPRPD056 treatments, and the tumors were removed to analyze their sizes (Figure 5C). It was observed that treatment with CpG-2722 or BPRDP056 alone suppressed tumor growth. Combination treatment involving CpG-2722 and BPRDP056 demonstrated more effective tumor suppression than treatment with any of these two agents alone (Figure 5B-D). The collected tumors were fixed and sectioned; the tissue was stained with hematoxylin and eosin to examine the infiltrating leukocytes. The results revealed that CpG-2722 and BPRDP056 alone and in combination increased the amounts of infiltrating leukocytes in tumors (Figure 5E). These findings suggest that the treatments activated immune responses in the tumors.

### Promoting immune cell accumulation in the tumor microenvironment via CpG-2722 and BPRDP056 treatment

**Figure 6:**
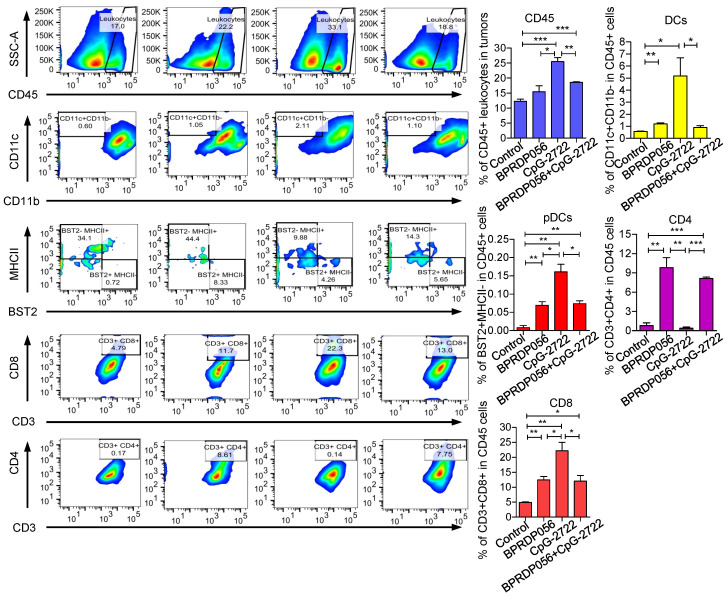
** CpG-2722 or BPRDP056 alone and in combination promote the accumulation of immune cells in head and neck squamous cell carcinoma (HNSCC).** Tumor-bearing mice treated with CpG-2722 or BPRDP056 alone or in combination in the experiment in **Figure 5** were euthanized at the end point to collect tumor samples. Tumor cells were dissociated and stained with surface-antibody for different immune cells as indicated. The immune cells composition was acquired on a FACS Canto II and analyzed using FlowJo software. Left panel: a representative set of histograms. Right panel bar figures: Data represent the mean ± SEM (n = 5). *, **, and *** represent statistically significant differences; *p* < 0.05, *p* < 0.01, and *p* < 0.001, respectively, compared with the control or as indicated.

The mechanism underlying the cooperative antitumor effect of CpG-2722 and BPRDP056 was further investigated. The accumulation of different immune cell types in tumors derived from different groups of mice treated with CpG-2722 or BPRDP056 alone and their combinations were investigated via flow cytometry. The results revealed that CpG-2722 induced the accumulation of CD45^+^ leukocytes, CD11c^+^ CD11b^+^ DC cells, BST2^+^MHCII^-^ pDC cells, and CD8^+^ T cells in tumors. BPRDP056 moderately induced CD11c^+^ CD11b^+^ DC cells, BST2^+^MHCII^-^ pDC cells, CD8^+^ T cells accumulation; however, it induced more CD4^+^ T cells in tumors compared with CpG-2722. Conversely, the profile of magnitude of immune cell accumulations induced by the combination of CpG-2722 and BPRDP056 was between that induced by either CpG-2722 or BPRDP056 alone (Figure 6), suggesting that the CpG-2722 played a major role in promoting immune cell accumulation in tumors. The infiltration of immune cells in tumors was further investigated via RT-PCR as this method is more sensitive and can detect more cell types in tumors. For the DC subsets, the results showed that BST2^+^ pDCs increased in the tumors of mice treated with CpG-2722 alone and combination of CpG-2722 and BPRDP056; CD205^+^, Clec9a^+^, and CD86^+^ cDC1s increased in the tumors of mice treated with CpG-2722 or BPRDP056 alone and their combination; however, the levels of Clec10a^+^ and SIRPα^+^ cDC2s did not change in the tumors of mice treated with any of these treatments (Figure S1A). For the macrophage subsets, treatment with CpG-2722 alone and combination of CpG-2722 and BPRDP056 promoted F4/80^+^ macrophage and iNOS^+^ M1 macrophage accumulation in tumors; conversely, no treatment affected the level of ARG-1 M2 macrophages in the tumor (Figure S1B). Additionally, treatment with BPRDP056 alone and combination of CpG-2722 and BPRDP056 promoted NKp46^+^ NK cell accumulation in tumors, and BPRDP056 alone treatment increased CD20^+^ B cell accumulation in tumors. Conversely, the levels of Ly6G^+^ granulocytes remained unaffected by the different treatments (Figure S1C). For the T-cell subsets, CD45^+^ leukocytes and CD3^+^ and CD8^+^ T cells were detected in the tumors of mice treated with CpG-2722 alone and combination of CpG-2722 and BPRDP056; in contrast, CD4+ T-cell accumulation in tumors was promoted via BPRDP056 alone treatment and not via the other treatments (Figure S1D). These results together with those obtained from flow cytometric analysis indicate that CpG-2722 plays a significant role in promoting immune cell accumulation in the tumors.

### Inducing cytokine production and PS exposure in tumors via CpG-2722 and BPRDP056 treatments

**Figure 7:**
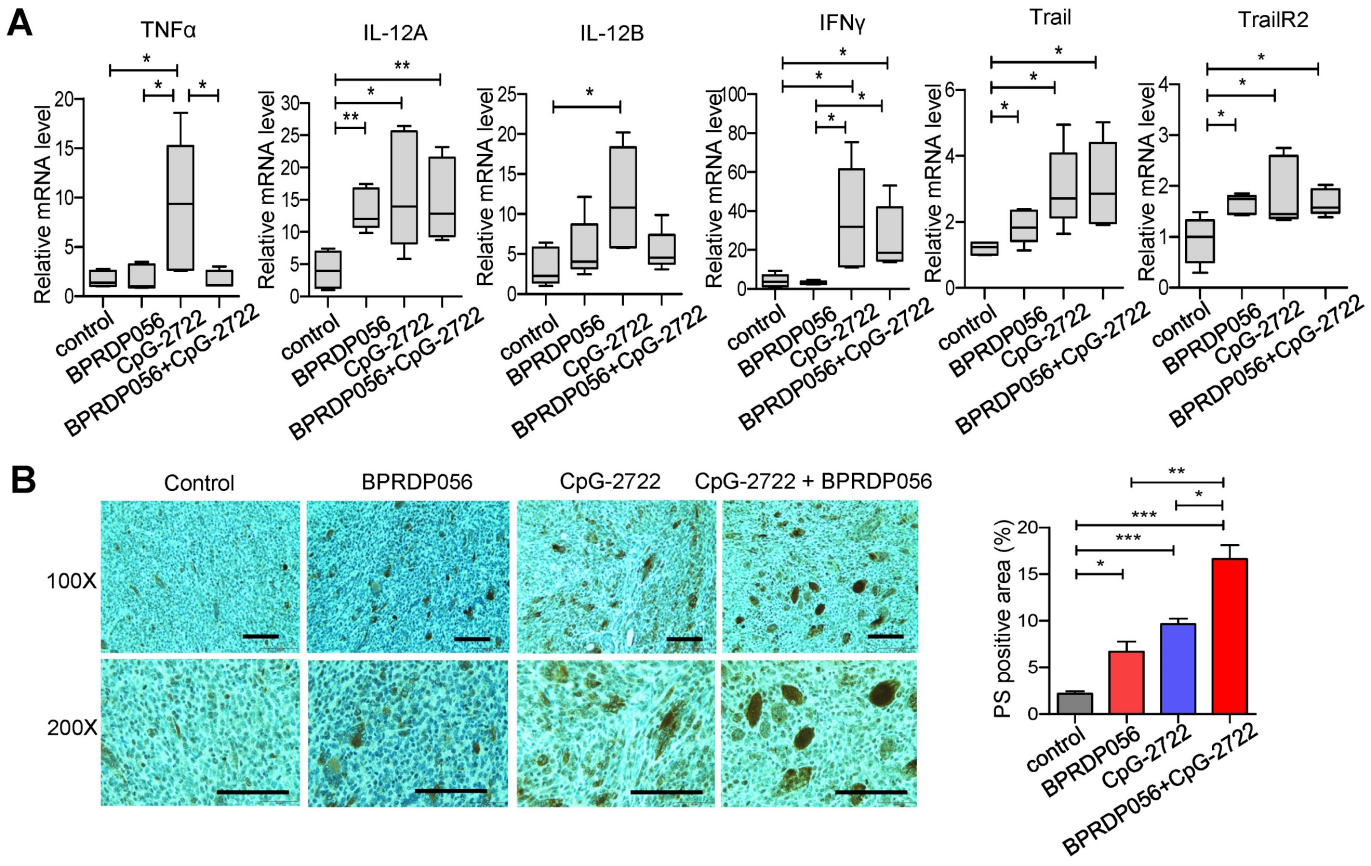
** CpG-2722 or BPRDP056 alone and in combination promote cytokine expression and phosphatidylserine exposure in head and neck squamous cell carcinomas (HNSCC).** Tumor-bearing mice treated with CpG-2722 or BPRDP056 alone or in combination in the experiment in **Figure 5** were euthanized at the end point to collect tumor samples. **(A)** Total RNA samples from the tumors were isolated by Trizol reagent. Expressions of different cytokines were analyzed by RT-qPCR. The expression level of β-actin was used as a loading control. **(B)** Tissue sections were immunohistochemically stained to detect PS exposure in tumors (upper left panel: 100× and bottom left panel: 200×). Scale bar represents 100 μm. PS exposure was quantified using Image J (right panel). *, **, and *** represent statistically significant differences; *p* < 0.05, *p* < 0.01, and *p* < 0.001, respectively, compared with the control.

Cytokines, including IL-12 and IFN-γ, play a crucial role in maintaining a favorable microenvironment for cancer immune therapy 38. Thus, the induction of cytokine expressions in the tumors of mice treated with CpG-2722 and BPRDP056 alone and their combination was investigated. RT-qPCR analyses revealed that treatment with CpG-2722 alone and combination of CpG-2722 and BPRDP056 promoted the expression levels of IL-12A, IFN-γ, Trail, and Trail receptor 2 (TrailR2) in tumors. CpG-2722 alone and not the other treatments activated TNF-α and IL-12 B expressions, and BPRDP056 alone increased IL-12A, Trail, and TrailR2 expressions in tumors (Figure 7A). The level of PS exposure in tumor is crucial for targeting BPRD056 to the tumor, therefore the effects of CpG-2722 and BPRDP056 treatments on PS exposure in tumors were investigated. The sections of tumors from the different groups of mice were stained using an anti-PS antibody. Treatment with CpG-2722 or BPRDP056 alone increased PS exposure in tumors, and their combination further enhanced PS exposure (Figure 7B).

### CpG-2722 increases PS exposure in cancer cells by inducing cytokine release from immune cells

**Figure 8:**
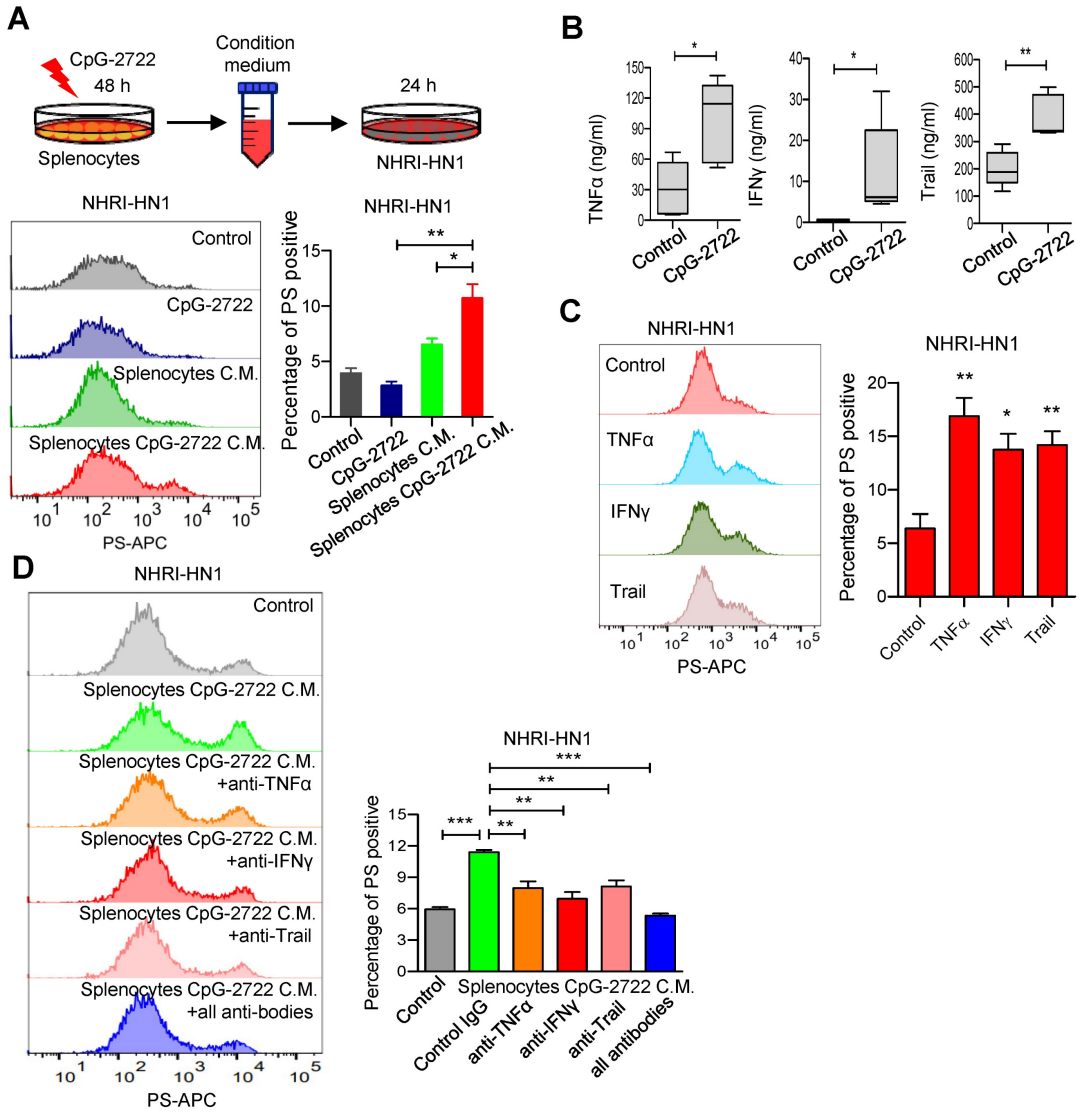
** CpG-2722 treatment increases PS exposure on cancer cells through induction of cytokines from immune cells. (A)** Mouse splenocytes were stimulated with/without 5 μg/ml of CpG-2722 for 48 h, and collected the condition medium (C.M.). NHRI-HN1 cells were treatment with/without 5 μg/ml of CpG-2722 or with/without medium containing 25% C.M. for 24 h as illustrated in the top panel. Phosphatidylserine (PS) exposure on the NHRI-HN1 cells were stained by annexin V conjugated APC antibody, and acquired on a FACS Canto II and analyzed using FlowJo software. Bottom left panel: a representative set of histograms. Bottom right panel: Data represent the mean ± SEM (n = 3). **(B)** Cytokine levels of TNF-α, IFN-γ, and Trail in tumors from the experiment in **Figure 5** were measured by ELISA. Data represent the mean ± SEM (n = 5). **(C)** NHRI-HN1 cells were treated with 100 ng/ml of TNF-α, IFN-γ, or Trail for 24 h. Levels of PS exposure were stained by annexin V conjugated APC antibody and acquired on a FACS Canto II. Left panel: a representative set of histograms. Right panel: Data represent the mean ± SEM (n = 3). **(D)** Mouse splenocytes were stimulated with/without 5 μg/ml of CpG-2722 for 48 h, and the C.M. was collected. NHRI-HN1 cells were incubated with medium containing 25% of the C.M. in the presence of 1 μg/ml of neutralizing antibody to TNF-α, IFN-γ, Trail or their combination as indicated for 24 h. Phosphatidylserine (PS) exposure on the NHRI-HN1 cells were stained by annexin V conjugated APC antibody, and acquired on a FACS Canto II and analyzed using FlowJo software. Bottom left panel: a representative set of histograms. Bottom right panel: Data represent the mean ± SEM (n = 3). *, **, and *** represent statistically significant differences *p* < 0.05, *p* < 0.01, and *p* < 0.001, respectively, compared with the control or as indicated.

BPRDP056 reportedly causes cancer cell death and PS exposure owing to its cytotoxicity 28. Conversely, PS exposure in tumors due to CpG-ODN treatment is unexpected and to the best of our knowledge, has not yet been reported. Therefore, the mechanism underlying this function of CpG-2722 was further investigated. Whether CpG-2722 directly activated PS exposure in cancer cells or indirectly activated the exposure via immune cell-mediated mechanisms was first investigated. The NHRI-HN1 cancer cells were incubated with/without CpG-2722 or incubated with condition media from splenocytes treated with/without CpG-2722 and analyzed via flow cytometry. The results revealed that the condition media from the CpG-2722-stimulated splenocytes showed activated PS exposure in cancer cells. Conversely, direct treatment with CpG-2722 did not induce PS exposure in cancer cells (Figure 8A). In addition to their proinflammatory activity, cytokines such as TNF-α, IFN-γ, and Trail have always been reported to increase PS exposure in cancer cells 39, 40. CpG-2722 induced TNF-α and IFN-γ expressions in splenocytes (Figure 3A). TNF-α, IFN-γ, and Trail induction in the tumors of the mouse groups subjected to different treatments was measured via ELISA. CpG-2722 alone treatment increased TNF-α, IFN-γ, and Trail protein production, consistent with the increased expression of these genes in the tumors of CpG-2722-treated mice (Figures 7A and 8B). PS exposure induction in cancer cells via these cytokines was verified by the treatment of the NHRI-HN1 cancer cells with TNF-α, IFN-γ, and Trail and analysis via flow cytometry (Figure 8C). Further, PS exposure due to the treatment of cancer cells with the condition media of CpG-2722-activated splenocytes was blocked via neutralizing antibodies against TNF-α, IFN-γ, and Trail or their combination (Figure 8D). These results suggest that CpG-2722 can induce PS exposure in tumors by inducing TNF-α, IFN-γ, and Trail production from immune cells.

**Figure 9:**
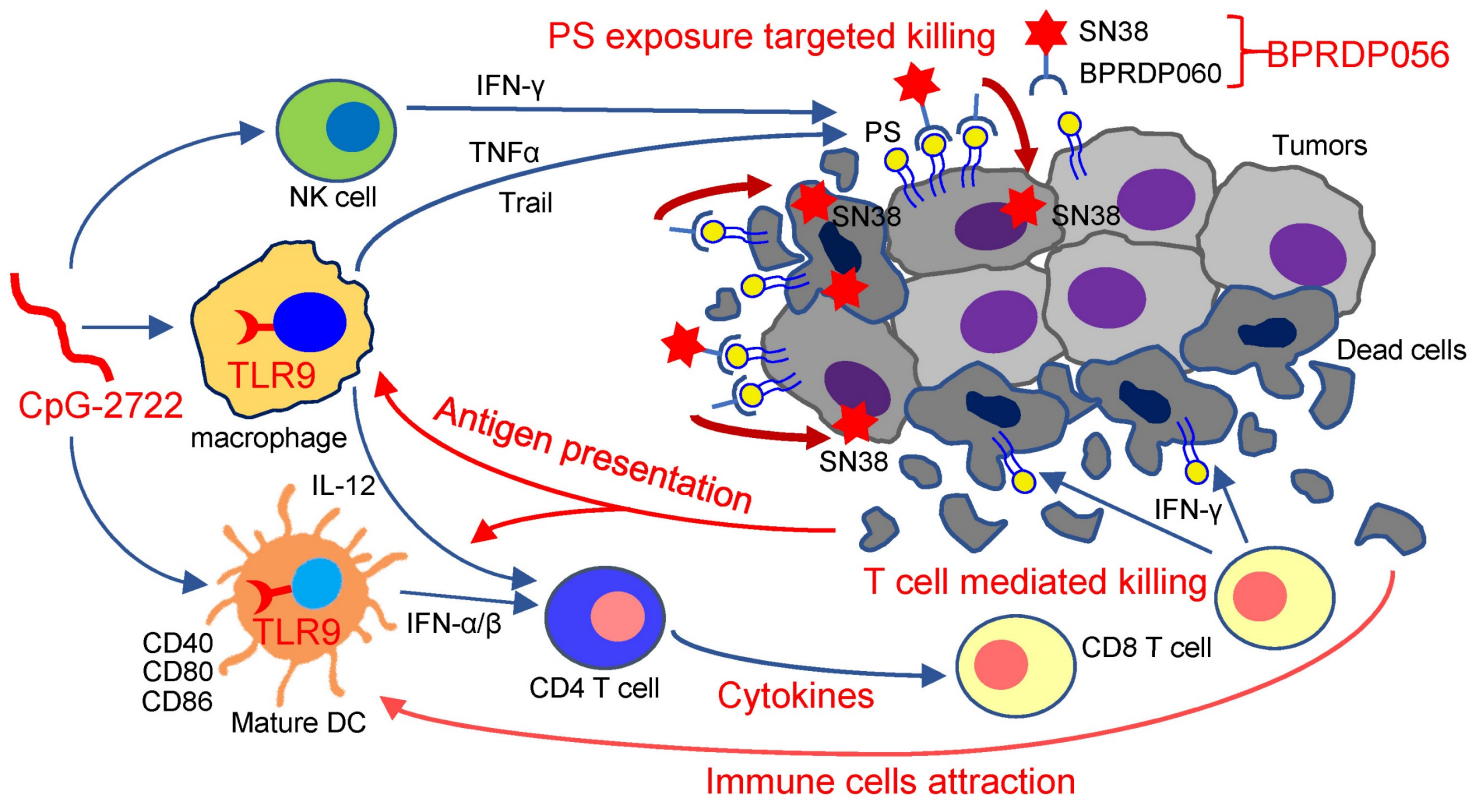
** Cooperative antitumor effects of CpG-2722 and BPRDP056.** In the tumors, TLR9 activation by CpG-2722 induces immune responses, including cytokine production and immune cell accumulation to increase T cell-mediated tumor killing. Conversely, BPRDP056 directly exhibits a cytotoxic effect on cancer cells. Additionally, TLR9 activation leads to increased phosphatidylserine (PS) exposure in cancer cells through inducing the production of TNF-α, IFN-γ, and Trail. This exposure attracts more BPRDP056 to the tumor site for cancer cell killing. Killed cells further expose PS in tumor for BPRDP056 targeting. Antigens released from the dead cells can be taken up by antigen-presenting cells, thereby enhancing the T cell-mediated tumor-killing effect promoted by CpG-2722. Thus, the actions of CpG-2722 and BPRDP056 form a positive feed-forward antitumor effect, and contribute to the cooperative antitumor effect of CpG-2722 and BPRDP056.

Thus, as illustrated in Figure 9, the results of this study indicate that the TLR9 activator, CpG-2722, increases immune cell accumulation and induces inflammatory cytokine expression in tumors, resulting in a favorable microenvironment for tumor eradication via the immune system. Additionally, CpG-2722 can promote PS exposure in tumors by inducing cytokines, such as TNF-α, IFN-γ, and Trail, from the immune cells to facilitate the PS targeting of BPRDP056 to release its payload SN38 in tumors. These account for the observed cooperative antitumor effect of CpG-2722 and BPRDP056.

## Discussion

Head and neck cancers are a group of malignancies comprising oral, oropharyngeal, nasopharyngeal, hypopharyngeal, laryngeal, sinus, salivary gland, and thyroid cancers. Alcohol consumption and smoking constitute the major causes of these cancers. Other risk factors include human papillomavirus infections and betel nut consumption. These cancers are a common cancer type worldwide. In the USA, they accounted for 66,000 cases and 15,000 deaths in 2022. Globally, they account for >660,000 new cases and 325,000 deaths annually. In addition to being life threatening, head and neck cancers severely impact the quality of life of patients and their families 1-3. Therefore, therapeutic agents and strategies are continuously developed. Herein, we utilized an NHRI-NH1 cell line-based syngeneic orthotopic head and neck cancer animal model to investigate the efficacy and functional mechanism of a combinational therapy involving TLR9 activator (CpG-2722) and PS-targeting cytotoxic prodrug (BPRDP056). The tumor-bearing mice were treated via intratumoral administration, as this route reportedly increases the antitumor effect of CpG-ODN, and cancer therapies with intratumoral injection of CpG-ODN are currently being intensively investigated in clinical trials. Furthermore, the development of image-guided injection techniques has rendered intratumoral injection in different organs feasible 41-44. We observed a cooperative antitumor effect between the CpG-2722 and BPRDP056.

NHRI-NH1 is one of the few cell lines available for establishing a syngeneic head and neck cancer animal model. Others include the TC-1, MOC1/2, and 4MOSC cell lines. Nevertheless, TC-1 was derived from primary lung cells via immortalization with the retroviral transduction of HPV16 E6/E740, and the MOC1/2 cell lines were generated from gene-deficient mice 45, 46. 4MOSC is a 4-nitroquinoline 1-oxide (4NQO)-induced murine oral squamous cell line 47. Conversely, NHRI-NH1 cells are generated by enriching the stemness of M11-1-2 HNSCC cells, which were immortalized from 4-NQO/arecoline-induced tumors in C57BL/6J mice. The M11-1-2 cells can cause tumor growth in immune-deficient mice but cannot develop tumors in immune-competent mice 33. As NHRI-HN1 cells are stemness-enriched cancer cells, they are probably more malignant and resistant to BPRDP056 than M11-1-2 cells (Figure 2). The NHRI-HN1 cells showed gene expression and signaling profiles similar to those of human oral SCC tissue 33. Therefore, animal cancer models generated using this cell line are reliable for studying HNSCC.

The cooperative antitumor effect of CpG-2722 and BPRDP056 can arise from their distinct and complementary antitumor functions. CpG-2722 induced antitumor immune responses, and BPRDP056 directly exerted cytotoxicity toward cancer cells. CpG-ODNs can trigger TLR9-mediated innate immune responses, such as inducing the expression of costimulatory molecules and cytokine production in antigen-presenting cells. These responses facilitate Th1 immune responses, thereby expanding tumor-specific T cells for tumor-cell killing 14-17. In line with this, we observed the activity of CpG-2722 in the induction of DC maturation. Additionally, CpG-2722 induced the expression of various inflammatory cytokines in splenocytes. The type I IFN produced plays a crucial role in linking innate immune responses to T cell-mediated antitumor response 48, 49. IL-12 and IFN-γ are two cytokines critical for antitumor responses. IL-12 is produced chiefly by innate immune cells, including natural killer cells, DCs, monocytes, and macrophages, and play a significant role in inducing IFN-γ production from T cells. IFN-γ is a signature cytokine for the Th1 immune response. It regulates T-cell differentiation and activation 34, 50. Further, CpG-2722 induces immune cell accumulation, including that of pDCs, cDC1, M1 macrophages, and CD8 T cells, and activates cytokine production, including that of TNF-α, IL-12, and IFN-γ, in tumors. These processes create a tumor microenvironment favorable for antitumor immune responses.

The antitumor activity of BPRDP056 depends mainly on its cytotoxicity. BPRDP056 is a prodrug of SN38, a topoisomerase I inhibitor 27, 28. Topoisomerase I plays a crucial role in DNA replication and transcription and is essential for cell proliferation. Therefore, the cytotoxicity of topoisomerase I inhibitors mainly arises from their antiproliferative effects, and these inhibitors are antitumor drug candidates because cancer cells have a proliferation rate higher than that of normal cells 51, 52. A prodrug of SN38, irinotecan (also known as CPT-11), was approved for the treatment of non-Hodgkin's lymphoma, cervical cancer, small cell lung cancer, and colon cancer. Nevertheless, irinotecan causes adverse effects at a higher dose, thereby limiting its therapeutic usage 53. Therefore, BPRDP056 was designed using a linker to connect Zn-DAP with the cytotoxic SN38 for this drug candidate to have a PS-dependent tumor-targeting mechanism. This design reduces the cytotoxicity of SN38 by prohibiting the premature release of SN38 before reaching the tumor 28.

BPRDP056 induced immune responses in splenocytes and tumors, although the responses were milder than those induced by CpG-2722. The mechanism underlying the immunogenicity of BPRDP056 remains unclear. Topoisomerase I inhibitors, including topotecan, camptothecin, irinotecan, and SN38, reportedly induce immunogenic cell death, eliciting an immune response in tumors. In this process, exposure to damage-associated molecular patterns (DAMPs), such as high-mobility group box 1, ATP, and calreticulin, from the cells killed by these inhibitors promotes the recruitment of immune cells and production of cytokines in the tumor 54, 55. As BPRDP056 is a prodrug of SN38, it probably follows a similar mechanism to activate immune responses through SN38 release. The immunogenic cell death caused by BPRDP056 may be enhanced by decreased clearance of apoptotic cells due to the binding of its Zn-DAP part to the apoptotic cells. Generally, PS exposure facilitates apoptotic cell clearance via phagocytes. If apoptotic cells are not efficiently cleared, they may undergo necrosis and release inflammatory DAMPs. In this regard, previous studies have reported that blocking PS by annexin V inhibits apoptotic cancer cell clearance, initiates necrosis, and renders apoptotic cells immunogenic, resulting in increased antitumor response 56-58. Therefore, although the immunostimulatory effect of BPRDP056 is not as strong as that of CpG-2722, this effect may still contribute to the antitumor effect of this prodrug.

Cancer cells expose PS on their surface; therefore, PS is a marker for tumor diagnosis, imaging, and targeting for drug treatment 22, 23. Herein, we discovered a novel function and mechanism of TLR9 activation, which increased PS exposure in cancer cells. CpG-2722 induced the production of cytokines, including TNF-α, Trail, and IFN-γ, from immune cells, which in turn activated PS exposure on cancer cells. In addition to its ability to induce antitumor immune responses, PS exposure induction in tumors via CpG-2722 likely plays a pivotal role in the cooperative antitumor effects of CpG-2722 and BPRDP056. As BPRDP056 is designed to preferentially bind to PS on cancer cells, the function of CpG-2722 in increasing PS exposure in cancer cells would facilitate BPRDP056 targeting in these cells. Additionally, both BPRDP056 and its payload SN38 reportedly induce cancer cell apoptosis [Figure 7B, 28, 59, 60], leading to the accumulation of more PS in the tumor following BPRDP056 targeting. Thefore, the actions between CpG-2722 and BPRDP056 may cooperatively induce the PS exposure as that observed in Figure 7B to increase PS targeting involved in tumor killing.

Thus, this study found that combining TLR9 activator and PS-binding cytotoxic prodrug, CpG-2722 and BPRDP056, respectively, results in a cooperative antitumor effect. CpG-2722 triggers immune responses and PS exposure in tumors, while BPRDP056 releases SN38 to kill cancer cells. PS exposure in dying cancer cells attracts more BPRDP056 to the tumor site, and tumor antigens released from the dead cells are taken up by antigen-presenting cells, thereby enhancing the CpG-2722-promoted T cell-mediated tumor-killing effect. Together, CpG-2722 and BPRDP056 form a feed-forward mechanism that helps kill cancer cells more effectively. This study also suggests a novel strategy of utilizing the PS-inducing function of TLR9 agonists for combinational treatments. Future research can explore the potential of integrating this concept to facilitate various cancer diagnoses and treatment with PS-targeting drugs. This approach could also enable the development of novel PS-targeting drugs in a more promising direction.

## Supplementary Material

Supplementary figure and table.

## Abbreviations

PS: phosphatidylserine
HNSCC: Head and neck squamous cell carcinoma
TLR: Toll-like receptors
NF-κB: nuclear factor-κB
IRF: interferon regulatory factor
IFN: interferon
MyD88: myeloid differentiation primary response 88
IRAK: interleukin-1 receptor-associated kinase
TRAF6: tumor necrosis factor-associated factor 6
TAK1: transforming growth factor-β-associated kinase 1
TNF-α: tumor necrosis factor (TNF)-α
IL: interleukin
DC: dendritic cell
CpG-ODNs: CpG-oligodeoxynucleotides
Zn-DPA: Zinc (II)-dipicolylamine
Trail: TNF-related apoptosis-inducing ligand
ELISA: enzyme-linked immunosorbent assay
SCC: squamous cell carcinoma
BMDC: bone marrow-derived dendritic cell
Flt3-L: FMS-like tyrosine kinase 3 ligand
GM-CSF: granulocyte-macrophage colony-stimulating factor
TrailR2: Trail receptor 2
