# Supplementary material for: Induction of Immune Responses and Phosphatidylserine Exposure by TLR9 Activation Results in a Cooperative Antitumor Effect with a Phosphatidylserine-targeting Prodrug
Source: Int J Biol Sci. 2023 May 11;19(9):2648–62. doi: 10.7150/ijbs.81683 (PMC10266080; doi:10.7150/ijbs.81683)
Supplement: Supplementary file 1 — Supplementary figure and table. [file ijbsv19p2648s1.pdf]

Supplementary Tab. 1 PCR primers for mouse genes used in this study.

Gene	Sequence
β-Actin For	GCTACAGCTTCACCACCACA
β-Actin Rev	AAGGAAGGCTGGAAAAGAGC
GAPDH For	AGAACATCATCCCTGCATCC
GAPDH Rev	CACATTGGGGGTAGGAACAC
ARG-1 For	ACAGGGCTCCTTTCAGGACTA
ARG-1 Rev	AGTCAGTCCCTGGCTTATGGT
BST2 For	CAATCTACTTCGCCGTCACA
BST2 Rev	TCTTCTCCAGGGACTCCTGA
CD3 For	CTGCTACACACCAGCCTCAA
CD3 Rev	ATCAGCAAGCCCAGAGTGAT
CD4 For	CTGATGTGGAAGGCAGAGAAG
CD4 Rev	GAGACCTGGGGTATCTTGAGG
CD8 For	TATGGCTTCATCCCACAACA
CD8 Rev	GACTGGCACGACAGAACTGA
CD20 For	GTCTTCGCACCCATCTGTTT
CD20 Rev	AATGGCAGCAAAGAGGCTTA
CD45 For	TCAGCAAGTTTCCCATCAAAG
CD45 Rev	CCCTTGTGCAGCAATGTATTT
CD86 For	TCAGTGATCGCCAACTTCAG
CD86 Rev	TTAGGTTTCGGGTGACCTTG
CD205 For	GTTTGTTGCTGATCCCCACT
CD205 Rev	CACCCACCACTTCTGTTCCT
Clec9a For	CAGTGGGACATTCCTACCTCA
Clec9a Rev	AGACCACACAGGAAATCATCG
Clec10a For	GGATCCCAAAATTCCCAGTT
Clec10a Rev	CCCTCTTCTCCACTGTGCTC
F4/80 For	CTCTGTGGTCCCACCTTCAT
F4/80 Rev	GATGGCCAAGGATCTGAAAA
IL-1β For	AGAGCTTCAGGCAGGCAGTA
IL-1β Rev	AGGTGCTCATGTCCTCATCC
IL-6 For	CCGGAGAGGAGACTTCACAG
IL-6 Rev	TTTCCACGATTTCCCAGAGA
IL-12A For	ACGGCCAGAGAAAAACTGAA
IL-12A Rev	CTACCAAGGCACAGGGTCAT
IL-12B For	CACGCCTGAAGAAGATGACA
IL-12B Rev	AGTCCCTTTGGTCCAGTGTG
IFNα For	CTGCTGGCTGTGAGGAAATAC
IFNα Rev	AGACAGGGCTCTCCAGACTTC

IFNβ For	GCCTTTGCCATCCAAGAGATGC
IFNβ Rev	ACACTGTCTGCTGGTGGAGTTC
IFNγ For	CAGCAACAGCAAGGCGAAAAAGG
IFNγ Rev	TTTCCGCTTCCTGAGGCTGGAT
iNOS For	GCCCCTGGAAGTTTCTCTTC
iNOS Rev	TTCTGTGCTGTCCCAGTGAG
Ly6G For	TGTGCTCATCCTTCTTGTGG
Ly6G Rev	AGGGGCAGGTAGTTGTGTTG
Nkp46 For	ACTGTGCCTTGGGCTATGTC
NKp46 Rev	CTCGATGGCTTTGGTCTCTC
SIRPα For	AGGTAGCCCACATCACCTTG
SIRPα Rev	GGAGATCCTCGGGGTAGAAC
TNFα For	ACGGCATGGATCTCAAAGAC
TNFα Rev	GTGGGTGAGGAGCACGTAG
Trail For	CCCTGCTTGCAGGTTAAGAG
Trail Rev	GGCCTAAGGTCTTTCCATCC
TrailR2 For	GTCAGAAGGGAACTGCAAGC
TrailR2 Rev	GCATCGACACACCGTATTTG

Supplementary Fig 1

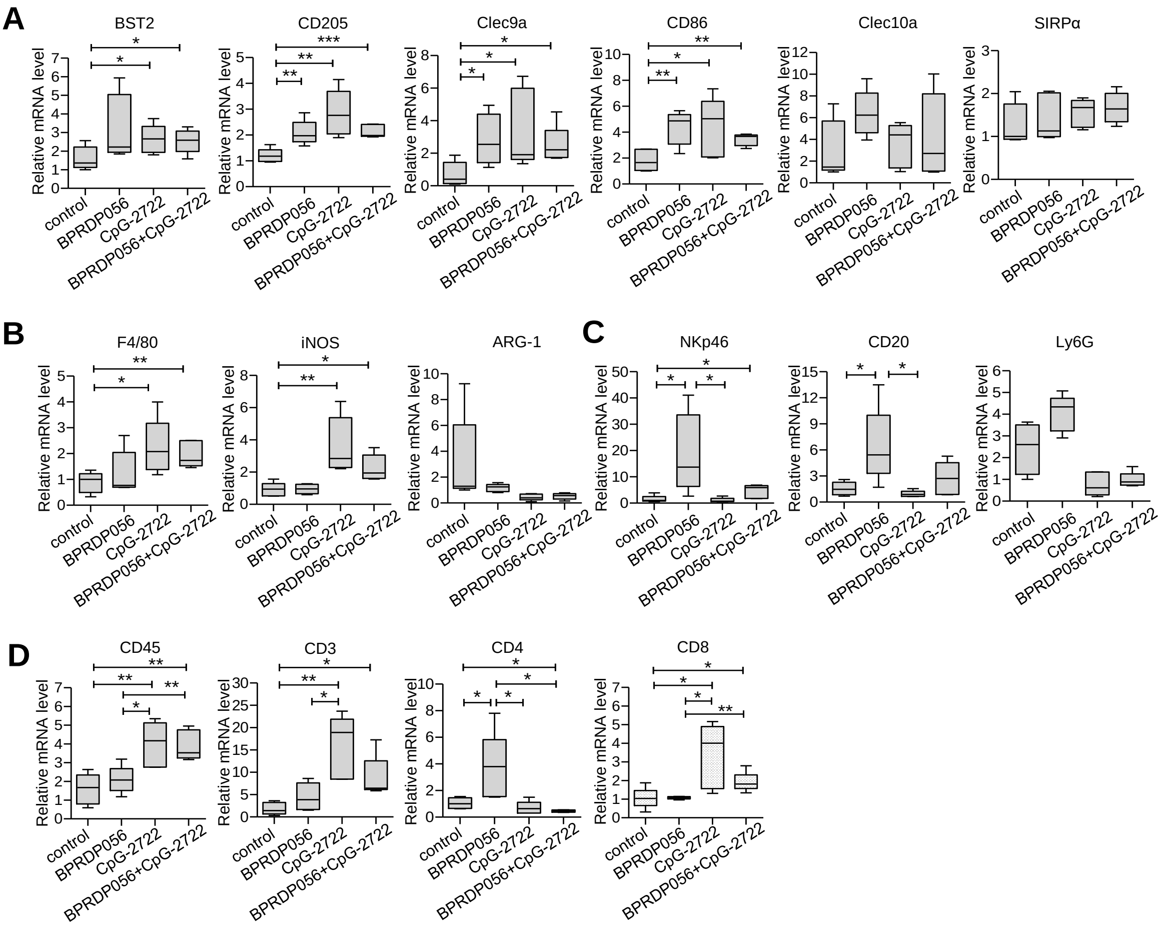

Supplementary Fig. 1 RT-qPCR analysis for the CpG-2722 or BPRDP056 alone and in combination promoted accumulation of immune cells in head and neck tumors. Tumor-bearing mice treated with CpG-2722 or BPRDP056 alone or in combination in the experiment in Fig. 5 were euthanized at the end point to collect tumor samples. Total RNA samples were isolated from the tumors with Trizol reagent. Expressions of markers for **a** different subsets of dendritic cells, **b** different subsets of macrophages, **c** natural killer cells, B cells, and neutrophils, and (D) different subsets of T cells were analyzed by RT-qPCR. The expression level of β-actin was used as a loading control. Data represent the mean ± SEM (n = 5). *, **, and *** represent statistically significant differences; $p < 0.05$, $p < 0.01$, and $p < 0.001$, respectively, compared with the control or as indicated.
